# Microsporidia Promote Host Mitochondrial Fragmentation by Modulating DRP1 Phosphorylation

**DOI:** 10.3390/ijms23147746

**Published:** 2022-07-13

**Authors:** Jian Luo, Jinzhi Xu, Chaolu Xie, Zuoming Zhao, Junrui Guo, Yuan Wen, Tian Li, Zeyang Zhou

**Affiliations:** 1 State Key Laboratory of Silkworm Genome Biology, Southwest University, Chongqing 400715, China; jianluo0214@163.com (J.L.); xjz_swu@126.com (J.X.); 13028325332@163.com (C.X.); zzmxndx@163.com (Z.Z.); 18790523552@163.com (J.G.); wy13102340311@163.com (Y.W.); 2 Chongqing Key Laboratory of Microsporidia Infection and Control, Southwest University, Chongqing 400715, China; 3 College of Life Science, Chongqing Normal University, Chongqing 400047, China

**Keywords:** microsporidia, mitochondrial fragmentation, DRP1, PGAM5, host–pathogen interaction

## Abstract

Microsporidia are obligate intracellular parasites that infect a wide variety of hosts ranging from invertebrates to vertebrates. These parasites have evolved strategies to directly hijack host mitochondria for manipulating host metabolism and immunity. However, the mechanism of microsporidia interacting with host mitochondria is unclear. In the present study, we show that microsporidian *Encephalitozoon* greatly induce host mitochondrial fragmentation (HMF) in multiple cells. We then reveal that the parasites promote the phosphorylation of dynamin 1-like protein (DRP1) at the 616th serine (Ser616), and dephosphorylation of the 637th serine (Ser637) by highly activating mitochondrial phosphoglycerate mutase 5 (PGAM5). These phosphorylation modifications result in the translocation of DRP1 from cytosol to the mitochondrial outer membrane, and finally lead to HMF. Furthermore, treatment with mitochondrial division inhibitor 1 (Mdivi1) significantly reduced microsporidian proliferation, indicating that the HMF are crucial for microsporidian replication. In summary, our findings reveal the mechanism that microsporidia manipulate HMF and provide references for further understanding the interactions between these ubiquitous pathogens with host mitochondria.

## 1. Introduction

Microsporidia are obligate intracellular parasites infecting nearly all invertebrates and vertebrates [1,2]. There are over 200 genera and 1400 species of microsporidia have been reported. Seventeen species of microsporidia have been found to infect humans, among which *Encephalitozoon cuniculi*, *Encephalitozoon hellem*, and *Encephalitozoon intestinalis* are the mostly reported and can cause extensive morbidity and mortality in immunocompromised and immune-deficient patients [3,4,5,6].

Microsporidia harbor extremely reduced genomes, and lack mitochondria and peroxisomes, leading to a high dependence on host cell-derived nutrients for proliferation [7,8,9]. Meanwhile, microsporidia have evolved the ability to exploit specific mechanisms to evade the cellular immune system by manipulating host mitochondria [10]. In early ultrastructural studies, microsporidian, including *E. cuniculi*, *E. hellem*, *Nosema disstriae*, and *Nosema* sp., appears to maximize ATP supply by the direct binding of mitochondria to the parasitophorous vacuole (PV) and meronts [11,12,13,14]. Simultaneously, *Hepatospora eriocheir* infection-induced host mitochondria were clustered around the plasmalemma of meronts [15]. Proteomic and quantitative real-time PCR analysis showed that the proteins and genes involved in oxidative phosphorylation and fatty acid metabolism were significantly up-regulated by *Nosema bombycis* infection [16,17]. Moreover, *E. hellem* interplay with host mitochondria through the sporoplasm surface protein 1 (EhSSP1), which interacts with voltage-dependent anion channels (VDACs) [14]. These studies suggested that microsporidia can further modulate host mitochondria, but the mechanisms have been rarely reported.

Mitochondria are dynamic for transforming between fusion and fission, which strongly affects the function of the organelle [18]. Mitochondrial dynamics is a highly regulated process that, when interrupted, can alter metabolism, proliferation, and apoptosis [19,20]. A variety of proteins play important roles in mitochondrial fusion and fission, such as optic atrophy 1 (OPA1) and mitofusins (MFN1 and MFN2) [19]. More specifically, dynamin 1-like protein (DRP1, also known as DNM1L) is a key protein regulating mitochondrial fission, which is a cytosolic protein that must translocate to mitochondria to induce fission [18]. DRP1 activation and translocation involve calcineurin-dependent DRP1 serine residue 637 (Ser637) dephosphorylation and DRP1 serine residue 616 (Ser616) phosphorylation [21,22]. Moreover, phosphoglycerate mutase 5 (PGAM5) has been reported to have a full-length and cleaved form, the last of which is active and required to dephosphorylate DRP1 to induce mitochondrial fission [23,24,25]. When mitochondria are fragmenting, DRP1 translocates from the cytosol to mitochondria, binding to its OMM receptor’s mitochondrial fission factor (MFF) or mitochondrial fission 1 protein (FIS1) [26].

Accordingly, intracellular pathogens can modulate host mitochondrial dynamics for survival and proliferation. For example, *Legionella pheumophila*, *Brucella*, and *Mycobacterium tuberculosis* induced host mitochondrial fragmentation (HMF) through secreting effector proteins [27,28,29]. *Chlamydia trachomatis* induced host mitochondrial elongation in early stages and HMF in late stages, depending on modifying the Ser637 of DRP1 [30]. By changing mitochondrial morphology, pathogens can manipulate host cell fate. For instance, *Helicobacter pylori* induced host cell apoptosis by activating DRP1 for promoting HMF [31]. *Salmonella enterica* inspired autophagy-mediated cell death of macrophages by distorting mitochondrial cristae morphology [32]. The intracellular microsporidia have been found to tightly contact with host mitochondria. However, it is unknown how these interactions affect the morphology of host mitochondria.

Among the microsporidia infecting humans, *E. cuniculi* and *E. hellem* are culturable and mostly used as model species in the laboratory. Therefore, by utilizing the two pathogens, we analyzed the morphological changes of host mitochondria during infection and revealed the induction of DRP1-dependent HMF.

## 2. Results

### 2.1. Microsporidian Infections Induced Drastic HMF in Different Cells

**Figure 1:**
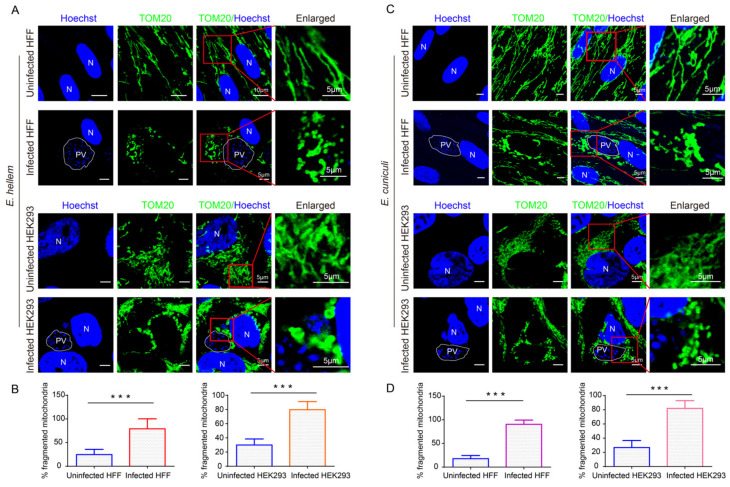
Analysis of host mitochondrial morphology in *Encephalitozoon*-infected cells at 48 hpi. (**A**,**C**) Mitochondrial morphology was detected in *E. hellem*- and *E. cuniculi*-infected HFF and HEK293 cells, respectively. Laser confocal shows nucleus labeled with Hoechst (blue). Mitochondria are labeled with the antibody against TOM20 (green). Bar, 5 μm; PV, parasitophorous vacuole; N, nucleus. (**B**) The percentage of mitochondrial fragmentation in the *E. hellem*-infected HFF and HEK293 cells. (**D**) The percentage of mitochondrial fragmentation in the *E. cuniculi*-infected HFF and HEK293 cells. The punctate mitochondrion was identified as a fragmented one. A total of 100 cells were counted for three replicates with at least 30 cells for each replicate. *** *p* < 0.001.

By confocal fluorescence microscopy, we firstly showed the dramatic changes on mitochondrial morphology in *E. hellem*-infected HFF and HEK293 cells (Figure 1A). As a result, we found that significant HMF occurred in infected cells (Figure 1B). Additionally, the prominent HMF was also observed in *E. cuniculi*-infected HFF and HEK293 cells (Figure 1C,D). Moreover, HMF was also significantly promoted in *E. hellem*- and *E. cuniculi*-infected DC2.4 cells (Figure S1). These findings suggest that Encephalitozoon infection induces HMF. 

**Figure 2:**
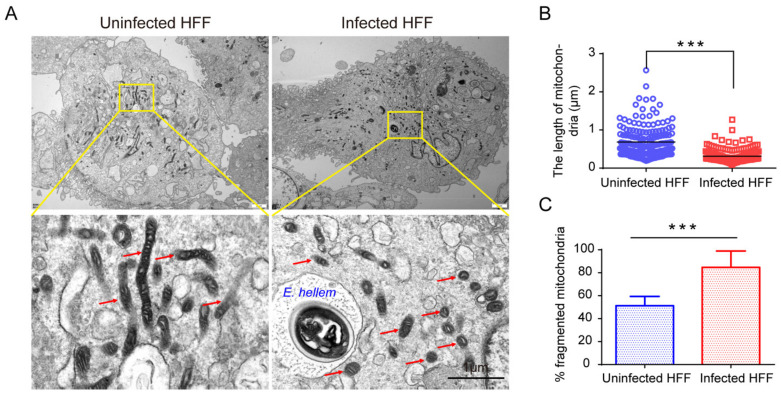
Observation of the HMF by transmission electron microscopy (TEM) in infected cells at 48 hpi. (**A**) TEM shows the HMF (red arrows) in *E. hellem*-infected HFF cells. The yellow box shows an enlarged view. Bar, 1 μm. (**B**) Determination of the length changes of mitochondria. At least 150 mitochondria from 10 TEM photographs for each sample were examined. Data of at least three separate experiments were expressed as a scatter plot. (**C**) The percentage of HMF in the uninfected and infected HFF cells. Twelve random cells from 10 TEM photographs for each sample were examined. *** *p* < 0.001.

**Figure 3:**
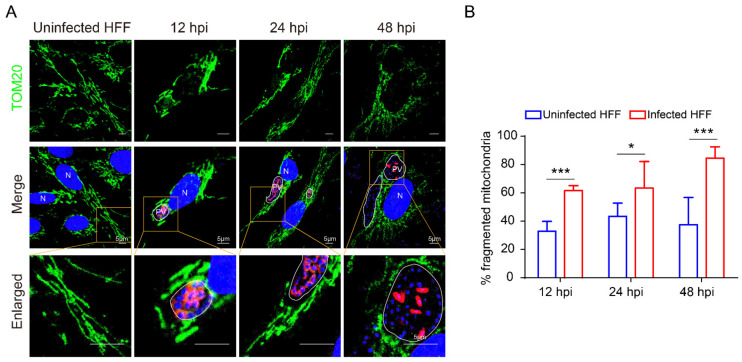
Analysis of the HMF in early and late stages of infection. (**A**) Observation of the mitochondrial morphology in *E. hellem*-infected HFF cells at 12, 24, and 48 hpi. Laser confocal shows nucleus labeled with Hoechst (blue), mitochondria labeled with anti-TOM20 antibody (green), and *E. hellem* meront labeled with FISH probe (red). The yellow box shows an enlarged view. Bar, 5 μm. PV, parasitophorous vacuole; N, nucleus. (**B**) Comparison of the HMF percentage between infected and uninfected HFF cells. At least 10 cells for each separate experiment were counted. * *p* < 0.05; *** *p* < 0.001.

To confirm the induction of HMF, we further studied the mitochondrial morphology in infected cells by transmission electron microscopy. As a result, significant HMF was confirmed by counting the length of mitochondria and the percentage of fragmentated mitochondria (Figure 2). Furthermore, we labeled *E. hellem* meronts in infected cells using the FISH probes and observed the mitochondria morphology at 12, 24, and 48 hpi, respectively. The FISH results confirmed significant HMF in infected cells (Figure 3A). Meanwhile, the percentage of HMF increased over infection time (Figure 3B).

### 2.2. Microsporidian Infections Promoted DRP1 Translocation to Mitochondria

**Figure 4:**
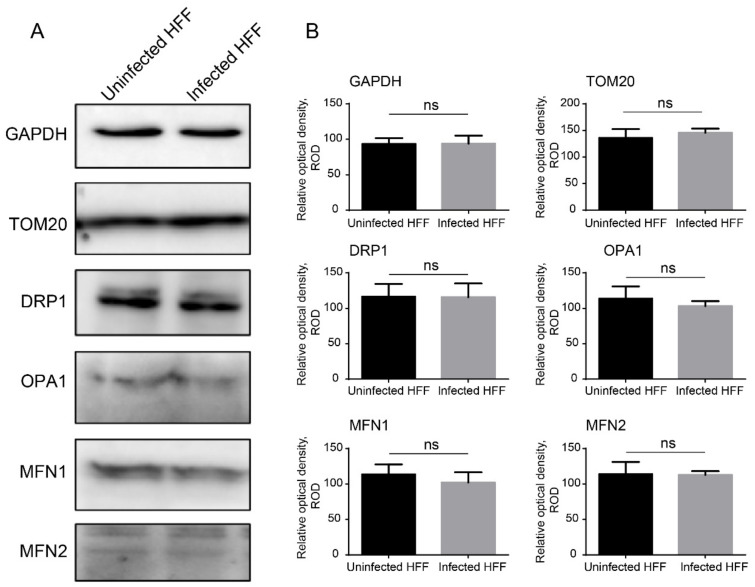
Characterization of DRP1 expression in host cells at 48 hpi. (**A**) *E. hellem*-infected HFF cells were analyzed by Western blotting to determine the regulators involved in mitochondrial dynamics, including DRP1, OPA1, MFN1, MFN2, and TOM20. GAPDH was used as an internal control. DRP1, dynamin 1-like protein; OPA1, optic atrophy 1; MFN1, mitofusin 1; MFN2, mitofusin 2; TOM20, translocase of the outer membrane of mitochondria 20; GAPDH, glyceraldehyde-3-phosphate dehydrogenase. (**B**) Statistical analysis of the relative optical density of Western blot bands. ns, not significant.

**Figure 5:**
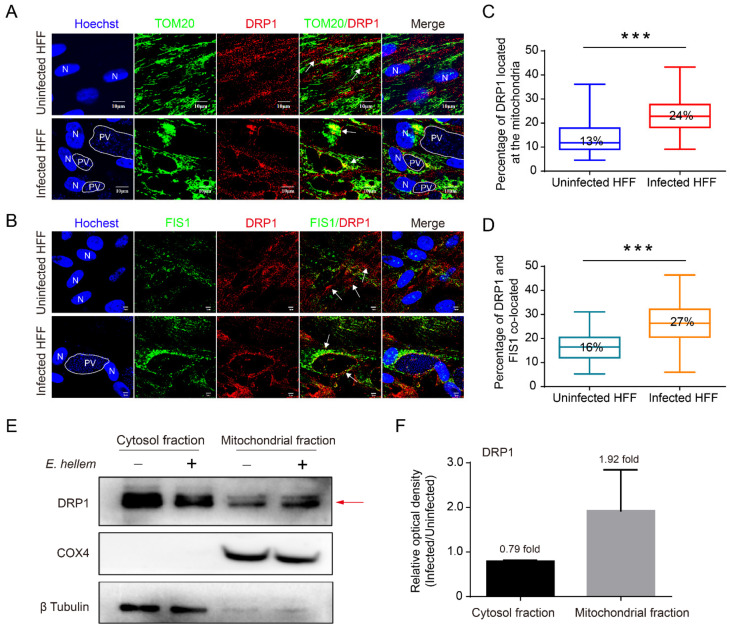
Subcellular localization of DRP1 in *E. hellem*-infected HFF cells at 48 hpi. (**A**,**B**) Observations of the subcellular localization of DRP1 by laser scanning confocal microscope. Nuclei are labeled with Hoechst (blue); DRP1 (red), TOM20 (green), and FIS1 (green) are labeled with relevant antibody, respectively. The white arrow indicates the co-localization of DRP1 with TOM20 and FIS1. PV, parasitophorous vacuole; N, nucleus. Statistical analysis of the co-localization of DRP1 with TOM20 (**C**) and FIS1 (**D**). At least 30 cells were counted for each sample. Data of at least three separate experiments are expressed as boxplots. *** *p* < 0.001. (**E**) The localization of DRP1 in cytoplasm and mitochondria. Cytoplasm and mitochondria fraction of 48 h infected HFF cells were isolated. COX4 and β Tubulin was used as an internal control. Red arrow shows the protein band with significant changes. (**F**) Comparison of the relative optical density of DRP1 immune blots between infected and uninfected cells.

Mitochondrial fusion and fission are essential for cell growth. The human protein DRP1, which is a large GTPase of the dynamin family, is a critical player in the process of mitochondrial fission [33]. We firstly evaluated the expression level of mediators involved in mitochondrial fission and fusion by Western blot (Figure 4). No significant change was found for DRP1, OPA1, MFN1, and MFN2 in *E. hellem*-infected HFF cells. We then evaluated the percentages of DRP1 located in the mitochondria through the co-immunostaining of DRP1 with TOM20 and FIS1 (a known DRP1 receptor). The proportion of DRP1 co-localized with TOM20 (24 ± 0.08%) and FIS1 (27 ± 0.09%) significantly increased in the infected cells (Figure 5C,D), suggesting that DRP1 was recruited to the mitochondria and induced mitochondrial fragmentation. This finding was also confirmed by the Western blot analysis of DRP1 in cytoplasm and mitochondrial fractions, the results of which show that DRP1 increases 1.92 ± 0.75-fold in the mitochondrial fraction of infected cells (Figure 5E,F).

### 2.3. Microsporidia-Induced HMF Depends on DRP1 Phosphorylation

**Figure 6:**
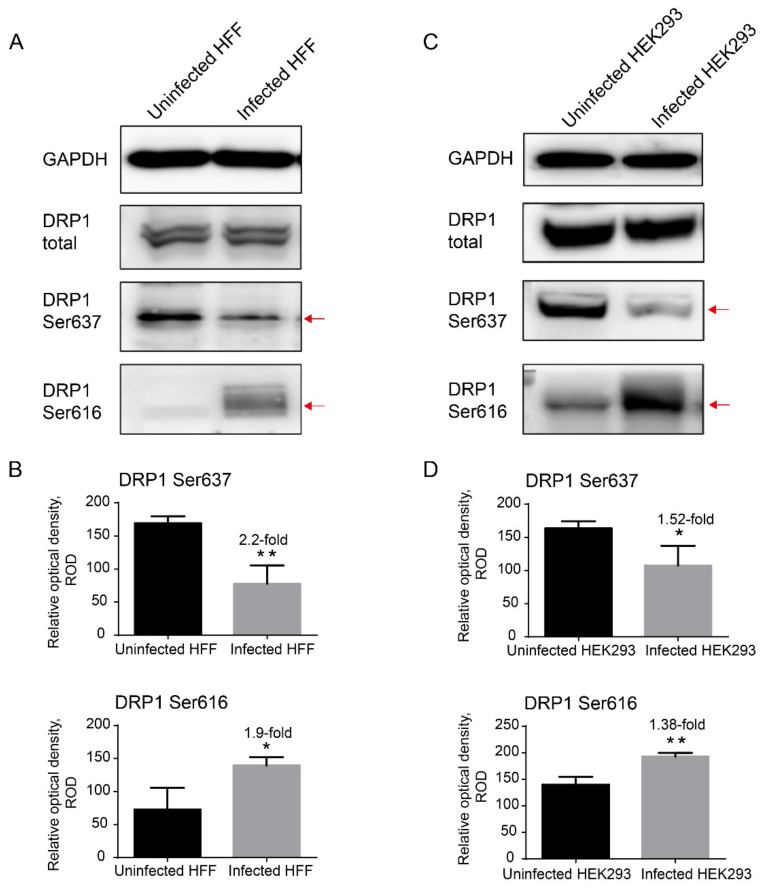
The regulation of DRP1 phosphorylation by *E. hellem*. (**A**,**C**) Western blot analysis of phosphorylation of DRP1 Ser637 and Ser616 in HFF cells ((**A**) the second replicate shown in Figure S4) and HEK293 cells ((**C**) the second replicate shown in Figure S4) 48 h post-infection by *E. hellem*, respectively. The GAPDH was used as an internal control. Red arrows indicate the significant changes in DRP1 phosphorylation. (**B**,**D**) Determination and statistical analysis of the relative optical density of DRP1 Ser637 and Ser616 calculated from the replicates of Western blot shown in Figure S4. * *p* < 0.05; ** *p* < 0.01.

It was reported that the translocation of DRP1 to mitochondria requires the phosphorylation of the 616th serine (Ser616) and dephosphorylation of the 637th serine (Ser637) [22]. Therefore, we verified the phosphorylation of DRP1 in microsporidia-infected HFF and HEK293 cells. As a result, we found a significant decrease in Ser637 phosphorylation and increase in Ser616 phosphorylation (Figure 6).

### 2.4. Microsporidia Infection Activated PGAM5 for Dephosphorylating the DRP1 Ser637

**Figure 7:**
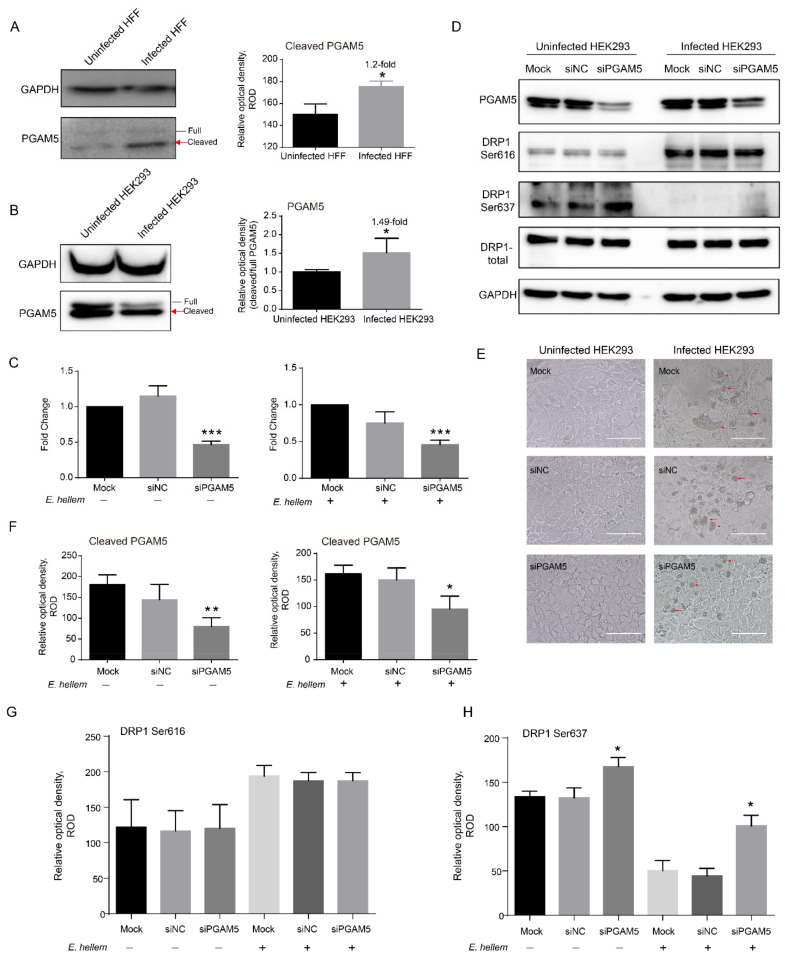
The activation of PGAM5 by *E. hellem*. Western blot analysis of the activation of PGAM5 in *E. hellem*-infected HFF (**A**) and HEK293 (**B**) cells at 48 hpi, respectively. GAPDH was used as an internal control. Red arrows mark the cleaved PGAM5 bands with significant changes. The relative optical density of cleaved PGAM5 bands was determined and statistically compared. (**C**) Transcript level of *PGAM5*. *GAPDH* used as an internal control gene. Mock, no treatment; NC, Negative control. (**D**) Western blot analysis of the dephosphorylation and phosphorylation of DRP1 with knockdown PGAM5 at 36 h. Simultaneously, the light-observed HEK293 cells are shown in the lower panels (**E**). Arrows indicate the PV of *E. hellem*. Bar, 50 μm. (**F**) The relative optical density of cleaved PGAM5 immune blots. (**G**) Statistical analysis of the relative optical density of immune blots for DRP1 Ser616. (**H**) Statistical analysis of the relative optical density of immune blots for DRP1 Ser637. * *p* < 0.05; ** *p* < 0.01; *** *p* < 0.001.

It has been proven that PGAM5 is required by the dephosphorylation of DRP1 Ser637 during mitochondrial fission [23,24,25]. In *E. hellem*-infected HFF and HEK293 cells, we determined the significant activation of PGAM5 by Western blot (Figure 7A,B). Furthermore, by knocking down the expression of PGAM5 using RNAi (Figure 7C–F), we found that the dephosphorylation of DRP1 Ser637 significantly decreased, and the phosphorylation of DRP1 Ser616 showed no significant change (Figure 7G,H). These results confirm that the dephosphorylation of DRP1 Ser637 requires the activation of PGAM5 during microsporidian infection.

### 2.5. Microsporidia Proliferation Required HMF

**Figure 8:**
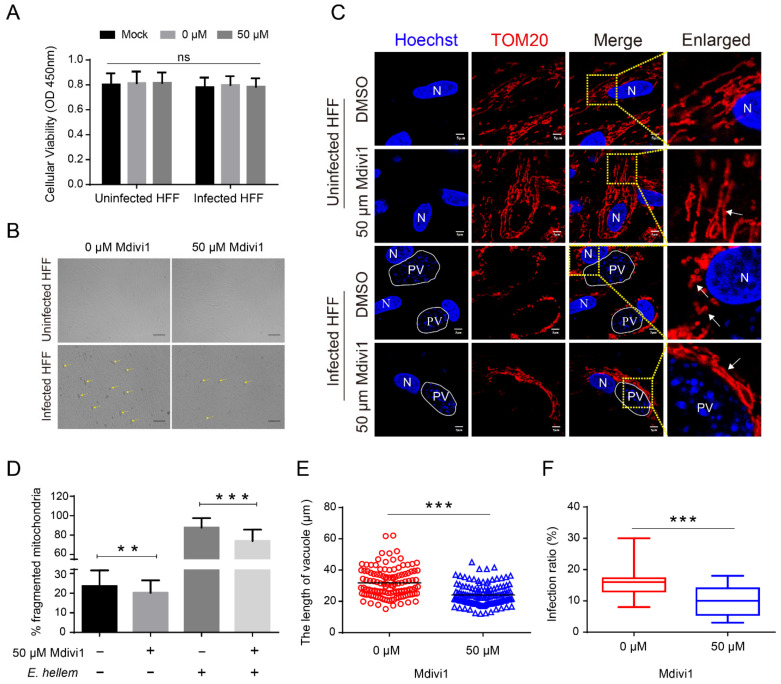
Analysis of the proliferation of microsporidia after inhibiting HMF at 48 hpi. HFF cells were treated with 50 μM Mdivi1 for 4 h, and then Mdivi1 was removed by the replacement of the whole medium. The cells were then infected by *E. hellem* and again treated with Mdivi1 after 24 hpi. (**A**) Determination of cell viability upon Mdivi1 treatment. Mock, normal cells without treatment; 0 μM Mdivi1, treatment by same volume of dimethyl sulfoxide (DMSO); (**B**) HFF cells infected by *E. hellem* at 48 hpi observed by light microscopy. Arrows indicate the PV of *E. hellem*. Bar, 100 μm. (**C**) Observations of HMF in *E. hellem*-infected and uninfected HFF cells treated with Mdivi1. The nucleus is labeled with Hoechst (blue). Mitochondria were stained with antibody against TOM20 (red). PV, parasitophorous vacuole; N, nucleus; Bar, 5 μm. (**D**) The percentages of HMF. A total of 100 cells were counted for three replicates with at least 30 cells for each replicate. (**E**) Analysis of the length of PV at 48 hpi after Mdivi1 treatment. At least 100 PVs from 10 fields for each sample were examined. Data of at least three separate experiments are expressed as scatter plots. (**F**) Statistical analysis of the infection rates in Mdivi1-treated cells. At least 10 fields for each sample were counted. ** *p* < 0.01, *** *p* < 0.001.

To address the role of HMF during microsporidia proliferation, we used Mdivi1 to specifically inhibit the DRP1 to suppress the mitochondrial fission. We firstly assessed the viability and morphology of cells treated with Mdivi1 and found no significant change (Figure 8A,B). When observing the mitochondrial morphology, we found a significant inhibition of HMF in infected cells and Mdivi1-treated cells (Figure 8C,D). Furthermore, we measured the size of PV and the infection rate at 48 hpi, and found that DRP1 inhibition significantly reduced the pathogen load (Figure 8B,E,F). These results strongly suggest that HMF is vital to microsporidian proliferation.

## 3. Discussion

Although it was reported that mitochondria were associated with the development of microsporidia for over 20 years [11], the host mitochondrial changes and factors involved remain unknown. In the present study, we demonstrated that microsporidia induced the significant dephosphorylation of Ser637 and phosphorylation of Ser616 in DRP1, resulting in mitochondrial fragmentation.

Ultrastructural studies have presented evidence that microsporidian PV and meronts directly bind to the host mitochondria to maximize the supply of ATP from the host [11,12]. It was also reported that *E. cuniculi* appears to maximize ATP supply by clustering ATP transporting porins VDACs to the vacuole–host mitochondria interaction site [12]. The knockout of VDACs significantly decreased the number and size of *E. hellem* PV [14]. Here, our work indicates that HMF is required by microsporidia for proliferation. These findings suggest that microsporidia-promoted HMF probably enhances the interactions between PV, and increases the host mitochondria and mitochondrial synthesis of ATP.

Studies show that accelerated mitochondrial division could decrease oxidative phosphorylation and ATP content and increase the oxidation of fatty acids and consume oxygen ratio (COR) [34,35]. However, the research also demonstrates that microsporidian infections upregulate the host expression of genes involved in ATP and promoted glycolysis and the TCA cycle, but remain at a normal level of ATP concentration [16,17,36]. These studies raise questions about why and how microsporidia upregulate host energy metabolism, but induce mitochondrial fragmentation. As shown in the studies on *L. pneumophila*, the pathogen can modulate host mitochondrial dynamics for upregulating mitochondrial oxidative phosphorylation (OXPHOS) and glycolysis at 1 hpi, but reduces OXPHOS and keeps glycolysis active at 6 hpi [27]. Therefore, it is possible that microsporidia could increase the host biosynthesis of nutrients and energy, but maintain a metabolic balance by promoting HMF.

It has been proposed that mitochondrial fragmentation would induce apoptosis [37]. Our data reveal that microsporidia induce HMF, but nucleus decay and the apoptotic body were not observed in the infected cells. Meanwhile, previous studies have shown that several species of microsporidia could suppress host apoptosis, including *E. cuniculi*, *N. bombycis*, and *Vittaforma corneae* [17,38,39,40,41]. This indicates that microsporidia can induce HMF while inhibiting host cell death. This regulatory mode was also found in infections by *L. pneumophila* and *Brucella* [27,28], suggesting a conserved strategy for pathogens modulating host mitochondria.

## 4. Materials and Methods

### 4.1. Cells and Reagents

Rabbit kidney epithelial (RK13) cell lines (CCL37, ATCC), human embryonic kidney 293 (HEK293) cell lines (CRL-1573, ATCC), and dendritic (DC2.4) cell lines (HTX2245, ATCC) were purchased from the American Type Culture Collection; and human foreskin fibroblast (HFF) cell lines were gifted by Dr. Han Bing from Shandong University, China, and grown in complete growth medium supplemented with 10% fetal bovine serum (10100154, Gibco, Thermo Fisher Scientific, Waltham, MA, USA) at 37 °C with 5% CO_2_. 

Anti-TOM20 rabbit monoclonal antibody (ab186735, Abcam, Cambridge, UK), anti-DRP1 mouse monoclonal (sc-101270, Santa Cruz, Dallas, TX, USA), anti-DRP1 (phospho Ser637) rabbit polyclonal (ab193216, Abcam), anti-DRP1 (phospho Ser616) rabbit polyclonal (#3455S, Cell Signalling Technology, MA, USA), anti-OPA1 mouse monoclonal (sc-393296, Santa Cruz), anti-MFN1 mouse monoclonal (sc-166644, Santa Cruz), anti-MFN2 mouse monoclonal (sc-515647, Santa Cruz), anti-GAPDH rabbit monoclonal (AF1186, Beyotime, Nantong, China), anti-β Tubulin mouse monoclonal (sc-166729, Santa Cruz), anti-COX4 rabbit monoclonal (ab202554, Abcam), anti-FIS1 rabbit monoclonal (ab156865, Abcam), anti-PGAM5 mouse monoclonal (sc-515880, Santa Cruz), and anti-PGAM5 rabbit monoclonal (ab126534, Abcam) were used. The following secondary antibodies were used: HRP-linked anti-mouse IgG antibody (BL001A, Bioshap, Hefei, China), HRP-linked anti-rabbit IgG antibody (BL003A, Bioshap), Alexa Fluor 488 goat anti-rabbit antibody (A32731, Invitrogen, Waltham, MA, USA), Alexa Fluor 488 goat anti-mouse antibody (16-240, Sigma, St. Louis, MO, USA), Alexa Fluor 647 goat anti-rabbit antibody (A32733, Invitrogen), and Alexa Fluor 647 goat anti-mouse antibody (A32728, Invitrogen). 

### 4.2. E. hellem and E. cuniculi Spore Preparation and Cell Infection

*E. hellem* and *E. cuniculi* spores were gifted by Dr. Han Bing from Shandong University, China, and cultured in RK13 cells, respectively. One week after infection, spores were purified from culture media by centrifugation in 70% Percoll (17089102, Cytiva, Marlborough, MA, USA) at 10,000× *g* for 15 min at room temperature (RT), then washed three times with sterile water, suspended in 0.5 mL of sterile distilled water, and stored at 4 °C until use [14,40,42]. The HEK293 and HFF cells were inoculated with spores by a cell-to-parasite ratio of 1:30, and all samplings of the infected cells were prepared 48 h post-infection (hpi).

An inhibitor of DRP1, Mdivi1 (ab144589, Abcam), was added to HFF cells at a 50 μM concentration for 4 h prior to infection, and then replaced the fresh complete medium, following a previous study [27], and it was added again after 24 hpi and treated for 24 h. Additionally, the same concentration of dimethyl sulfoxide (DMSO) (D8418, Sigma Aldrich) was added as a control. 

### 4.3. Indirect Immunofluorescence Assay (IFA)

Infected HFF and HEK293 cells were fixed with 4% paraformaldehyde for 10 min at RT, washed three times with PBS (phosphate buffer solution), and permeabilized in 0.1% Triton X-100 (PBS) for 15 min. Then, the cells were blocked in PBST (phosphate buffer saline with 0.05% Tween-20) containing 10% goat serum and 5% bovine serum albumin (BSA) for 1 h at RT. After being washed three times with PBS, the samples were incubated with anti-TOM20 rabbit antibody (diluted 1:500 in a blocking solution), anti-DRP1 mouse antibody (diluted 1:50 in a blocking solution), and anti-FIS1 rabbit antibody (diluted 1:200 in a blocking solution), for 8–12 h at 4 °C, respectively. After washing with PBST, the cells were incubated with Alexa Fluor 488 goat antibody and Alexa Fluor 647 goat antibody for 1 h at RT in the dark and washed three times with PBST. Both nuclei of the host cell and pathogen were stained with Hoechst 33342 (C1026, Beyotime, China) for 30 min at RT and washed three times. After staining, these samples were observed and photographed using an Olympus FV1200 laser scanning confocal microscope. 

With the photos, we measured the length of the PV and analyzed the co-localization of TOM20, FIS1, and DRP1 by ImageJ (https://imagej.nih.gov/ij/index.html, accessed on 8 June 2022). The length values were then used for the *t*-test with GraphPad Prism v6.01 (https://www.graphpad.com/scientific-software/prism/, accessed on 8 June 2022).

### 4.4. In Situ Fluorescence Hybridization (FISH)

Infected cells were prepared at 12, 24, and 48 hpi; fixed with 4% paraformaldehyde for 10 min; and permeabilized in 0.1% Triton X-100 (PBS) for 15 min, followed by incubation overnight at 46 °C with hybridization buffer (900 mM NaCl, 20 mM Tris [pH 7.5], 0.01% sodium dodecyl sulfate (SDS)) containing 5 ng/µL Cy3-ACTCTCACACTCACTTCAG (HPLC purified custom oligonucleotide, Sangon Biotech Co., Ltd. Shanghai, China) RNA probes [43,44], The infected cells were then washed at 48 °C for 1 h in wash buffer (900 mM NaCl, 20 mM Tris [pH 7.5], 0.01% SDS, 5 mM ethylenediaminetetraacetic acid) [45], and washed in PBS three times. Then, the cells were blocked, incubated with anti-TOM20 rabbit antibody and secondary antibody (Alexa Fluor 488 goat antibody), observed, and photographed using Olympus FV1200 laser scanning confocal microscopy. 

### 4.5. Transmission Electron Microscopy (TEM)

For TEM analysis, HFF cells were grown to confluence in T_25_ bottles and then infected with 1 × 10^6^ spores for 48 h. Uninfected and infected cells were fixed with 2.5% glutaraldehyde, postfixed with 1% osmium tetroxide for 2 h, and washed with 0.1 M PBS buffer. Then, the samples were dehydrated using an ascending acetone series and 100% acetone three times. They were infiltrated with gradient Epon812 resin (SPI, USA), sequentially embedded in absolute resin, and polymerized at 70 °C for 48 h. Ultrathin sections were cut on a Leica EM UC7 ultramicrotome, stained with 3% uranyl acetate 10–15 min, followed by lead citrate for 1–2 min, and photographed on a JEM-1400FLASH transmission electron microscope at an accelerating voltage of 80 kV.

### 4.6. Quantitative Analyses of HMF by Confocal Microscopy and TEM

Mitochondria were accurately labeled with anti-TOM20 in infected and uninfected cells. Fragmented mitochondria were identified, as previously described [46]. The punctate mitochondrion was identified as fragmented. To evaluate the morphological changes, we calculated the percentage of HMF for each cell. One hundred cells were manually counted for each sample. The overall experiment was independently repeated three times. Moreover, the changes in mitochondrial size were also analyzed by TEM. Twelve random cells from 10 TEM photos for each sample were examined. The length of the two ends of each mitochondrion was measured by ImageJ. The punctate mitochondrion was identified as fragmented. Mitochondrial numbers were manually counted. The rate of fragmentation was then calculated and analyzed using the GraphPad Prism for the *t*-test.

### 4.7. Western Blot Analyses of DRP1 Expression, Phosphorylation, and PGAM5 Activation

The uninfected and infected cells were seeded after 48 hpi, washed three times with PBS, and lysed in RIPA buffer (20 mM Tris, pH 7.5, 0.15 M NaCl, 1 mM EDTA, 0.5% Triton-X, 0.1% SDS) with a protease inhibitor cocktail (P1050, Beyotime, China) and phosphatase inhibitor cocktail (P1050, Beyotime, China). Cell lysates were centrifuged for 15 min at 12 000 g at 4 °C to sediment cell debris after 10 min incubation on ice. The total protein samples were separated by sodium dodecyl sulfate polyacrylamide gel electrophoresis (SDS-PAGE) and transferred to a polyvinylidene difluoride (PVDF) membrane (Roche). Membranes were blocked with 5% (w/v) nonfat dry milk (#9999, Cell Signalling Technology, Danvers, MA, USA) for 1 h at 37 °C, and incubated overnight at 4 °C with the primary antibody diluted in QuickBlock™ Primary Antibody Dilution Buffer (P0256, Beyotime, China), following three washes with TBST (Tris-buffered saline containing 0.05% Tween 20), and then 1 h at RT with the secondary antibody diluted in PBS. After three washes with TBST, the membrane was exposed with the ECL Western blot detection kit (#34580, Thermo Fisher Scientific) and imaged using an Azure Biosystems C300 imaging system. The relative optical density of Western blot bands was measured by ImageJ, values of which were analyzed using the GraphPad Prism for the *t*-test.

### 4.8. Western Blot Analyses of DRP1 Location

HFF cells were plated in two T_25_ bottles and grew to 90% confluency, and then infected with *E. hellem* for 48 h. A mitochondria isolation kit for profiling cultured cells (MITOISO2-1KT, Sigma Aldrich, USA) was used to isolate and purify mitochondria. Briefly, the uninfected and infected cells were collected and washed with ice-cold PBS for 5 min at 600 g at 4 °C. Resuspend the cell pellet in 1 mL of lysis buffer (cell lysis solution 1:200 (v/v) in 1× extraction buffer A containing a protease inhibitor cocktail), incubated on the ice for 5 min. Additionally, 2 mL 1× extraction buffer A was added. The lysates were centrifuged for 10 min at 1000× *g* at 4 °C to remove cell debris. The supernatant was collected and centrifuged for 10 min at 3500× *g* at 4 °C, and then the pellet/supernatant was the mitochondrial/cytosol fraction, respectively. The DRP1 location in the mitochondrial/cytosol protein was analyzed by Western blot. 

### 4.9. Analyses of Cell Viability

A Cell Counting Kit 8 (CCK-8) was used to detect the cell viability. A total of 10,000 cells per well were plated in 24-well plates. At the indicated time points, 30 μL of CCK-8 reagent (C0041, Beyotime, China) was added to the cells, according to the manufacturer’s protocol. Then, the cells were incubated for another 1 h at 37 °C with 5% CO_2_ and the optical density was measured at A450 nm.

### 4.10. RNAi of PGAM5

We knocked down the expression of PGAM5 by RNA interference (RNAi) with a siRNA, CCAUAGAGACCACCGAUAU, previously reported [47]. Negative control siRNA was obtained from Sangon Biotech (Shanghai) Co., Ltd. For RNA interference, 1 × 10^6^ HEK293 cells were plated in six-well cell culture plates, and the cells were transfected with 30 nM siRNA using INTERFERin siRNA Transfection Reagent (PT-409-01, Polyplus Transfection, Illkirch-Graffenstaden, France) and incubated for 36 h. Additionally, the same HEK293 cells were infected by *E. hellem* at 12 hpi, and then transfected and incubated with 30 nM siRNA for 36 h. The silencing efficiency of PGAM5 was evaluated using real-time quantitative PCR and Western blot assay. The RT-qPCR was conducted using PGAM5 primers (F: GCCGGAAGCTGTGCAGTATT; R: GGTGGGTGATGCTGCCATTA) and internal reference GAPDH primers (F: GAAGGTGAAGGTCGGAGTC; R: GAAGATGGTGATGGGATTTC).

## 5. Conclusions

**Figure 9:**
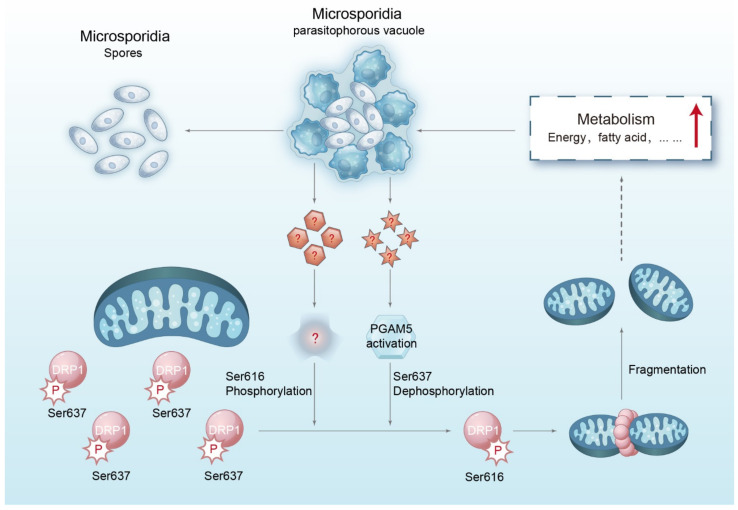
A model for microsporidia modulating HMF. Microsporidian infection promotes HMF by activating PGAM5, which dephosphorylates DRP1 Ser637. Moreover, the infection also increases the phosphorylation of DRP1 Ser616. Both modifications result in the translocation of DRP1 from cytosol onto the mitochondrial outer membrane, and mediates the fragmentation of mitochondria. The significant increase in HMF would up-regulate host metabolism and provide energy and nutrients for the proliferation of microsporidia. PGAM5, phosphoglycerate mutase 5; DRP1, dynamin 1-like protein.

In summary, we firstly reported the HMF during microsporidian infection, and revealed the mechanism of this modulation. We proposed a model (Figure 9) that the HMF induced by microsporidia depends on the dephosphorylation of DRP1 Ser637 and phosphorylation of DRP1 Ser616. Moreover, the HMF is required by microsporidia for proliferation. Our findings provide references for further understanding the interactions between microsporidia and host mitochondria.

## Data Availability

The data presented in this study are available on request from the corresponding author.

## Supplementary Materials

The following supporting information can be downloaded at: https://www.mdpi.com/article/10.3390/ijms23147746/s1.
