# Supplementary figures and images for: Microsporidia Promote Host Mitochondrial Fragmentation by Modulating DRP1 Phosphorylation
Source: Int J Mol Sci. 2022 Jul 13;23(14):7746. doi: 10.3390/ijms23147746 (PMC9321008; doi:10.3390/ijms23147746)

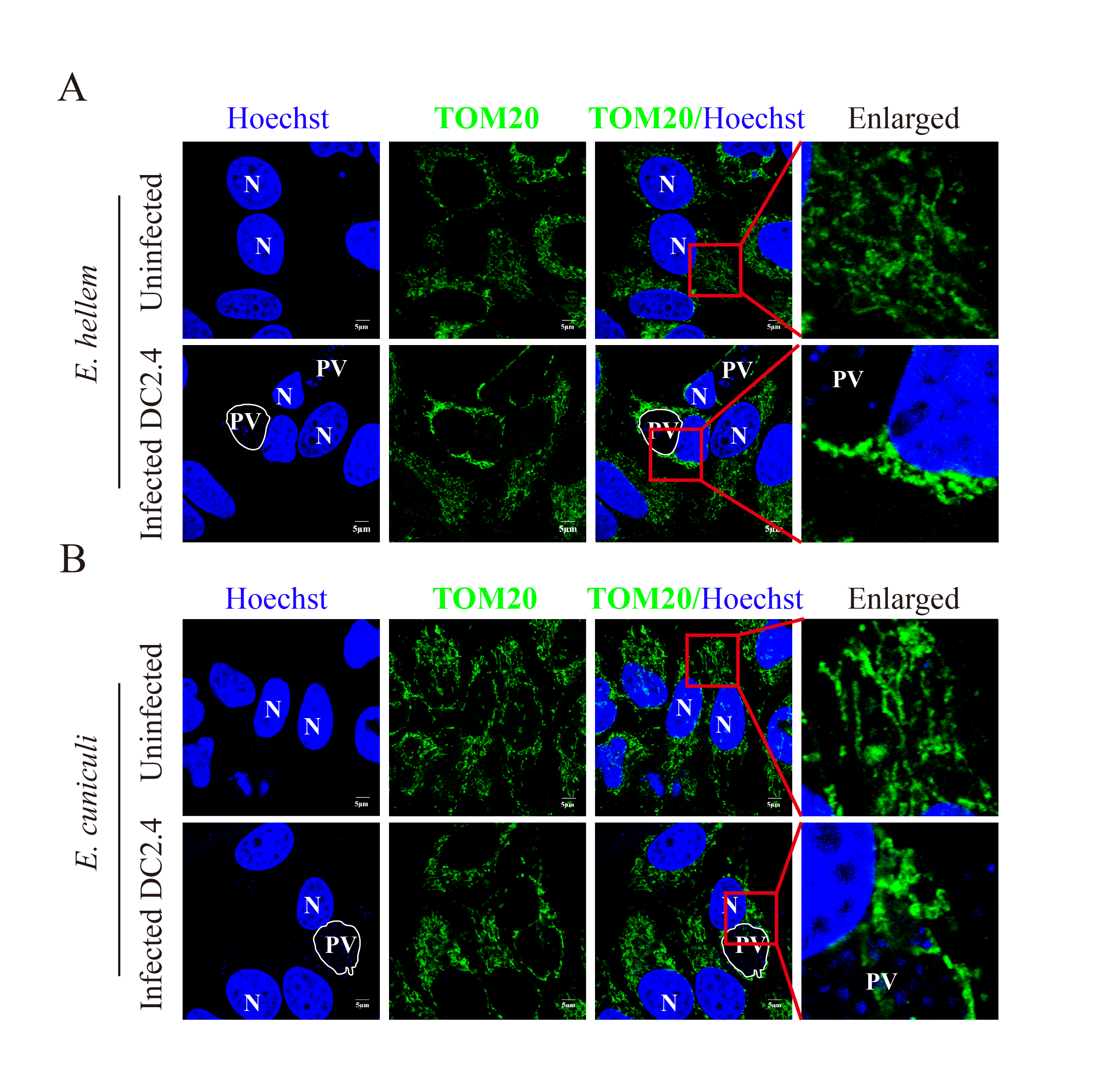

Supplement: Supplementary file 1 [file ijms-23-07746-s001.zip › FigureS1.tif]

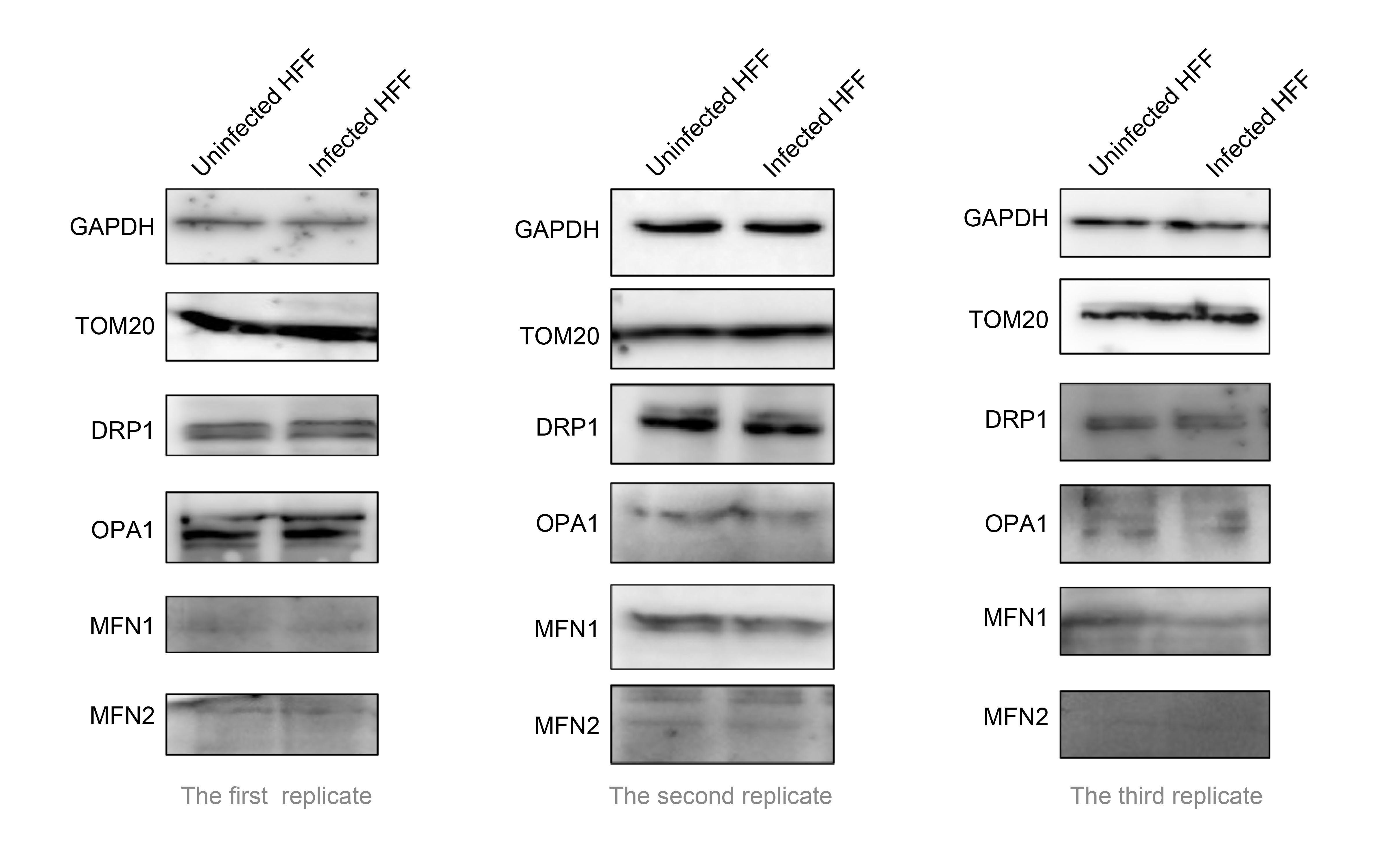

Supplement: Supplementary file 1 [file ijms-23-07746-s001.zip › FigureS2.tif]

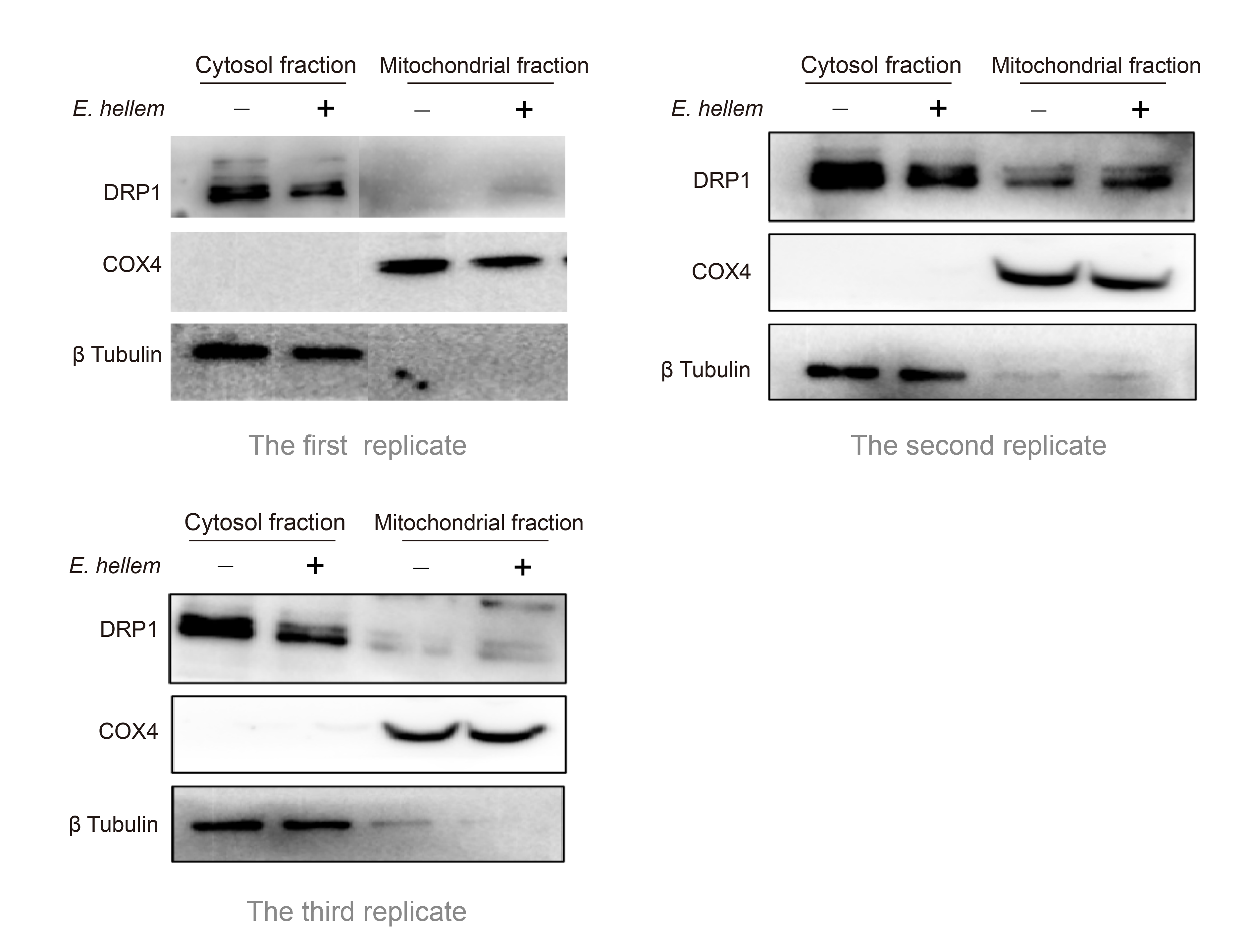

Supplement: Supplementary file 1 [file ijms-23-07746-s001.zip › FigureS3.tif]

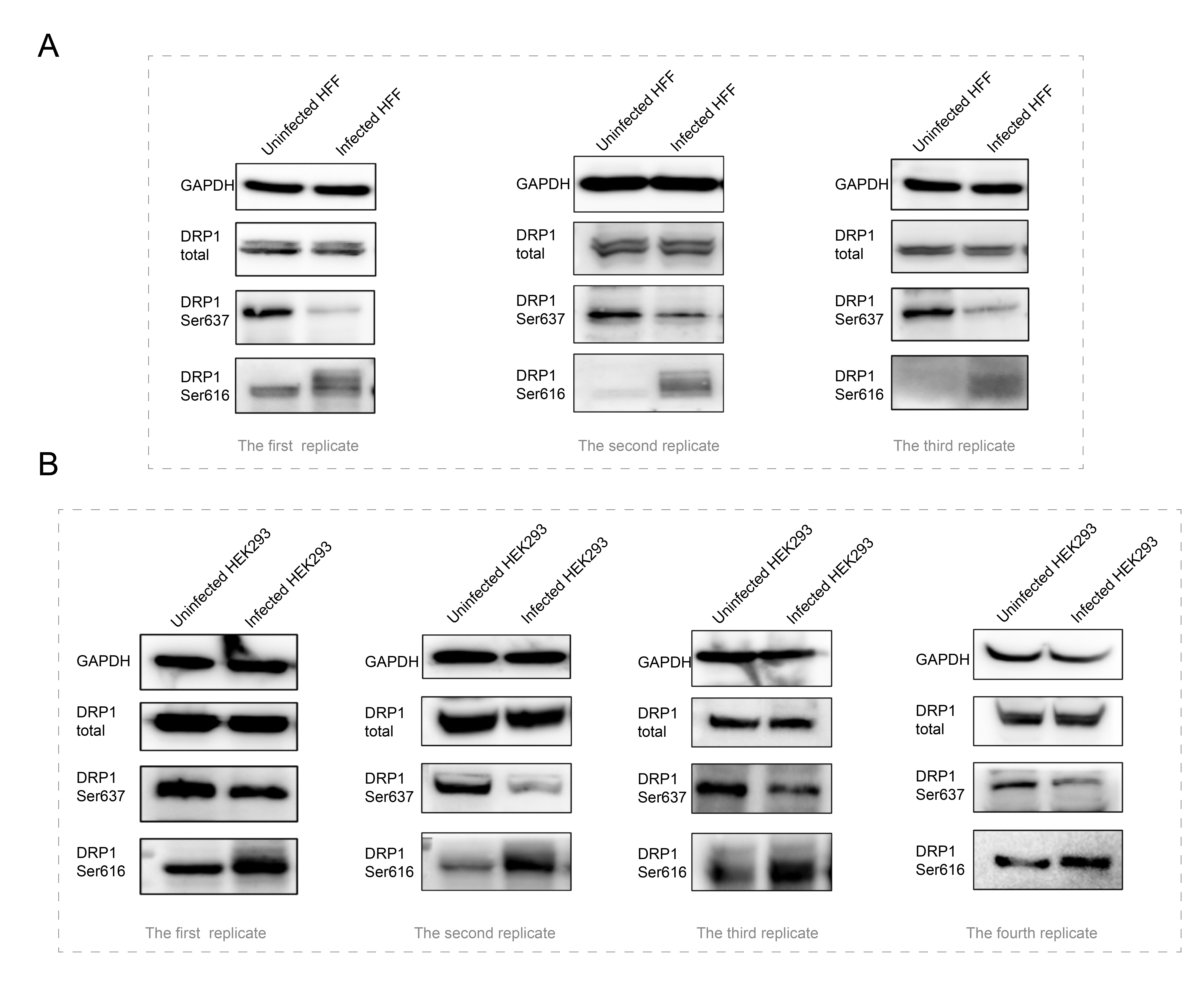

Supplement: Supplementary file 1 [file ijms-23-07746-s001.zip › FigureS4.tif]

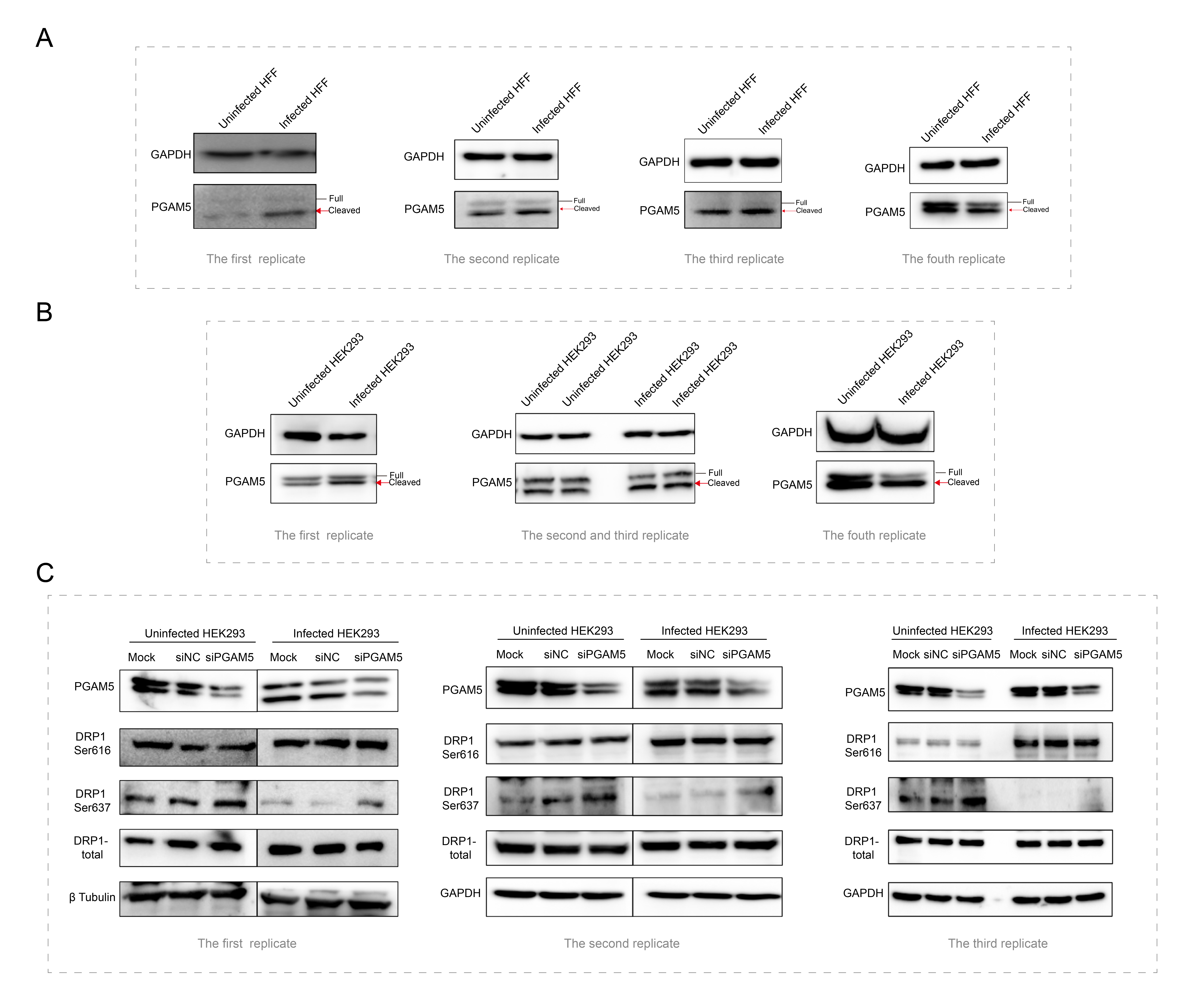

Supplement: Supplementary file 1 [file ijms-23-07746-s001.zip › FigureS5.tif]
